# Including random centre effects in design, analysis and presentation of multi-centre trials

**DOI:** 10.1186/s13063-021-05266-w

**Published:** 2021-05-22

**Authors:** Kate Edgar, Ian Roberts, Linda Sharples

**Affiliations:** 1 Department of Medical Statistics, LSHTM, Keppel Street, London, WC1E 7HT UK; 2 Clinical Trials Unit, LSHTM, Keppel Street, London, WC1E 7HT UK

**Keywords:** Multi-centre trials, Random effects, Heterogeneity

## Abstract

**Background:**

In large multicentre trials in diverse settings, there is uncertainty about the need to adjust for centre variation in design and analysis. A key distinction is the difference between variation in outcome (independent of treatment) and variation in treatment effect. Through re-analysis of the CRASH-2 trial (2010), this study clarifies when and how to use multi-level models for multicentre studies with binary outcomes.

**Methods:**

CRASH-2 randomised 20,127 trauma patients across 271 centres and 40 countries to either single-dose tranexamic acid or identical placebo, with all-cause death at 4 weeks the primary outcome. The trial data had a hierarchical structure, with patients nested in hospitals which in turn are nested within countries. Reanalysis of CRASH-2 trial data assessed treatment effect and both patient and centre level baseline covariates as fixed effects in logistic regression models. Random effects were included to assess where there was variation between countries, and between centres within countries, both in underlying risk of death and in treatment effect.

**Results:**

In CRASH-2, there was significant variation between countries and between centres in death at 4 weeks, but absolutely no differences between countries or centres in the effect of treatment. Average treatment effect was not altered after accounting for centre and country variation in this study.

**Conclusions:**

It is important to distinguish between underlying variation in outcomes and variation in treatment effects; the former is common but the latter is not. Stratifying randomisation by centre overcomes many statistical problems and including random intercepts in analysis may increase power and decrease bias in mean and standard error estimates.

**Trial registration:**

Current Controlled Trials ISRCTN86750102, ClinicalTrials.gov NCT00375258, and South African Clinical Trial Register DOH-27-0607-1919

**Supplementary Information:**

The online version contains supplementary material available at 10.1186/s13063-021-05266-w.

## Background

Large phase III randomised controlled trials (RCTs) often involve many centres, sometimes in several countries. Outcomes may vary, either due to centre differences, for example in infrastructure and concomitant treatment protocols, or due to environmental or nutritional variation in patient populations. For example, Papachristophi et al. showed that UK cardiac surgery outcomes vary substantially due to patient characteristics, operating surgeon and centre [1]. When analysing RCTs carried out in diverse settings, it is important to consider these sources of variation in outcome in order that the effect of the treatment of interest is estimated both accurately and precisely.

In primary trial analysis, important patient characteristics measured at baseline, especially those used to stratify randomisation, may be adjusted for in trial analysis and this has been shown to increase precision of the estimate for a normally distributed effect [2]. This has also been shown for survival data [3]. For moderately sized RCTs and where centre is not included in the randomisation process, failure to adjust for centre can cause biased standard errors of the estimated treatment effect, leading to incorrect type 1 error and a reduction in power [4]. For very large, stratified (by centre) trials, omitting centre from the primary analysis may be justified, since stratified randomisation will guard against bias in treatment effect estimates and the size of the trial will ensure adequate power. This was the case for the CRASH-2 placebo-controlled RCT of tranexamic acid in trauma patients, the main example in this study [5]. However, in discussing CRASH-2, Gruen et al. raised concerns about the efficacy of tranexamic acid for patients treated to modern trauma care standards, as CRASH-2 was carried out in mostly low- to middle-income countries and outcomes varied substantially [6]. It is not clear whether this concern surrounded variation in *treatment effect* or in the *underlying centre-specific outcomes*, and the difference between these two is often conflated.

Although randomisation in multicentre trials is often stratified by centre, including many centres as factors in the analysis can cause computational problems and make trial results difficult to interpret. One option is to include centres as random effects, whereby their results are viewed as a sample of centre outcomes and overall results pertain to the average [7]. A random effects model effectively reduces the number of parameters to be estimated and allows exploration of covariates acting at the country and centre levels, as well as the individual level. One study that used random effects models in this context found that large between-centre differences in outcomes did not affect the estimate or statistical significance of the overall treatment effect in a traumatic brain injury trial [8]. We aim to replicate this study using a larger trial and extend the methods in order to (i) demonstrate use and presentation of random effects for multicentre RCTs through reanalysis of the CRASH-2 RCT and (ii) clarify the implication for different RCT design options when the primary outcome is binary.

Specifically, we aim to:

- Assess variance in outcome, and treatment effect, between countries and between centres within countries, in the CRASH-2 trial;
- Clarify the interpretation of different sources of variation between centres and investigate centre-level covariates;
- Compare the treatment effect from an unadjusted analysis with that from analysis which accounts for between country and centre variance (using random effects);

## Methods

### Motivating example

The CRASH-2 trial, published in 2010 was a large, multicentre RCT comparing tranexamic acid with placebo in adult trauma patients at risk of significant bleeding [5]. The trial included 20,211 patients recruited from 274 hospitals in 40 countries, between May 2005 and January 2010. We focus on the primary outcome of death in hospital within 4 weeks of injury. In the trial, tranexamic acid significantly reduced all-cause mortality risk (relative risk 0.91, 95% CI 0.85 to 0.97; *p* = 0.0035). Patients were allocated to trial arm using a minimisation algorithm which ensured balance between the groups in age, sex, time since injury, type of injury (blunt or penetrating), Glasgow Coma Score, systolic blood pressure, respiratory rate, central capillary refill time and country. Although these factors were considered important predictors of outcome, the primary analysis was unadjusted and did not allow for differences in outcome or treatment effect between centres or between countries. Given the process of achieving balance across major predictive factors and the very large size of the trial, unadjusted analysis is justified. However, given the concerns of some investigators [6], a secondary analysis exploring centre and country differences is of interest.

### Analysis methods

(See Additional file 1 for details of statistical models). In contrast to the original analysis of CRASH-2, we used a *Binary logistic regression model*, since this is common for trial analysis and more suitable for small trials or uncommon outcomes. Ignoring centre and country effects, the log (odds) of death for a patient takes the form,

$$ {\beta}_0+{\beta}_1T+{\boldsymbol{\beta}}_{\mathbf{2}}^{\boldsymbol{T}}\boldsymbol{X} $$

where the intercept *β*_0_ is the log (odds) of death in the control (placebo) group with all covariates equal to zero, treatment coefficient *β*_1_ is the log (odds ratio) for the treatment group relative to control (all other factors being equal), *T* = 1 if treated and *T* = 0 if control, and ${\boldsymbol{\beta}}_{\mathbf{2}}^{\boldsymbol{T}}\boldsymbol{X}$ represents coefficients and values for all other variables in the analysis. In RCTs, ***X*** usually includes variables used in the assignment of patients to treatment groups. These baseline characteristics are included to remove systematic variation due to patient factors and to improve precision of the treatment effect.

### Random effects models

In the simple model above, the underlying probability of death and the effect of treatment may depend on patient characteristics but are otherwise constant across centres and countries. Equivalence of outcomes across centres may be unlikely when trials are conducted in diverse settings. One option is to allow for variation between centres using random effects. If variation in outcome is expected, a centre-specific term is added to the regression:

$$ {\beta}_0+{u}_{0i}+{\beta}_1T+{\boldsymbol{\beta}}_{\mathbf{2}}^{\boldsymbol{T}}\boldsymbol{X} $$

where *u*_0*i*_ denotes a random effect for centre *i*, which represents the difference in outcomes between each centre and the average outcome. The *u*_0*i*_ are usually assumed to vary according to a Normal distribution with mean zero. Allowing this kind of variation for the intercept (*β*_0_) illustrated by *u*_0*i*_ above represents the case where the underlying outcomes vary but the treatment effect is constant. We note that both trial arms are subject to this type of variation.

If variation in both outcome and treatment effect is expected, this adds another centre-specific term to the regression:

$$ {\beta}_0+{u}_{0i}+\left({\beta}_1+{u}_{1i}\right)T+{\boldsymbol{\beta}}_{\mathbf{2}}^{\boldsymbol{T}}\boldsymbol{X} $$

where *u*_1*i*_ denotes a random effect on the treatment coefficient for centre *i*, which again is assumed to be from a population of treatment effects, usually assumed to be normally distributed. Allowing centre variation in the treatment coefficient represents the case where the treatment affects patients differently in different centres. Although the intervention is only applied in one trial arm, it is the difference in outcomes between treated and controls that provides the data from which random treatment effects are calculated. In the above, two levels are assumed for the data, centres and patients within centres, but in practice, the data structure may be more complex. In CRASH-2, the data have a three-level hierarchical structure, countries, centres within countries and patients within centres. In this case, we include additional random effects representing country variation.

### Intra-class correlation coefficient (ICC)

The total variation in outcomes between patients, after adjusting for differences between patients, includes variation due to unexplained centre differences. Any bias and loss of power due to not accounting for between-centre variance depends on how correlated patients in the same centre are compared with patients from different centres. This is quantified by the intra-class correlation coefficient (ICC), which measures how much of the total variability is explained by the between-centre variance. If the ICC is one, outcomes for participants in the same centre are perfectly correlated, and if ICC is zero, outcomes for participants in the same centre have zero correlation. For three level hierarchies, both country and centre variation contributes to the total variation, so that both have an ICC.

#### Statistical software

We used Stata 15 software for these analyses.

## Results

### Trial data

Individual patient data from CRASH-2 were used [5] (summary statistics in Table 1). Four of 20,211 randomised patients withdrew their consent. Eighty patients with no outcome recorded and forty-two patients with one or more missing covariate (age, time from injury, Glasgow Coma Score (GCS) and systolic blood pressure (SBP)) were excluded. The remaining 20,085 patients were included in the all-cause mortality analysis. Because of the small number of exclusions, no additional analysis of missing data was undertaken. The number of patients entered into the trial by individual centres ranged from 1 to 1330, median 22.5 and interquartile range 64. The number of trial centres in each country ranged from 1 (13 countries) to 84, median 3 and interquartile range 5.25.

**Table 1:** Summary statistics for patient characteristics in the CRASH-2 trial

Patient level characteristics	Tranexamic acid (*n* = 10,060)	Placebo (*n* = 10,067)
Age (years)		
Mean (SD)	34.6 (14.14)	34.5 (14.39)
< 25	2777 (27.6%)	2838 (28.2%)
25–34	3006 (29.9%)	3070 (30.5%)
35–44	1966 (19.5%)	1832 (18.2%)
> 44	2311 (23.0%)	2326 (23.1%)
Sex		
Men	8413 (83.6%)	8456 (84.0%)
Women	1647 (16.4%)	1610 (15.99%)
Not known	0	1 (0.01%)
Glasgow Coma Scale (total)		
Severe (3–8)	1789 (17.8%)	1830 (18.2%)
Moderate (9–12)	1349 (13.4%)	1344 (13.4%)
Mild (13–15)	6915 (68.7%)	6877 (68.3%)
Not known	7 (< 1%)	16 (< 1%)
Time since injury (hours)		
Mean (SD)	3.22 (19.99)	3.26 (20.03)
≤ 1	3747 (37.3%)	3705 (36.8%)
> 1–3	3037 (30.2%)	2996 (29.8%)
> 3	3272 (32.5%)	3362 (33.4%)
Not known	4 (0.04%)	4 (0.04%)
Type of injury		
Blunt*	6788 (67.5%)	6817 (67.7%)
Penetrating	3272 (32.5%)	3250 (32.3%)
Systolic blood pressure (mm Hg)		
≤ 75	1562 (15.5%)	1599 (15.9%)
76–89	1609 (16.0%)	1689 (16.8%)
≥ 90	6878 (68.4%)	6761 (67.2%)
Not known	11 (< 1%)	18 (< 1%)
28-day mortality		
Dead (all-cause)	1463 (14.5%)	1613 (16.0%)
Dead due to bleeding	489 (4.9%)	575 (5.7%)

*Includes patients with both blunt and penetrating and those with only blunt injuries

**Fig. 1:**
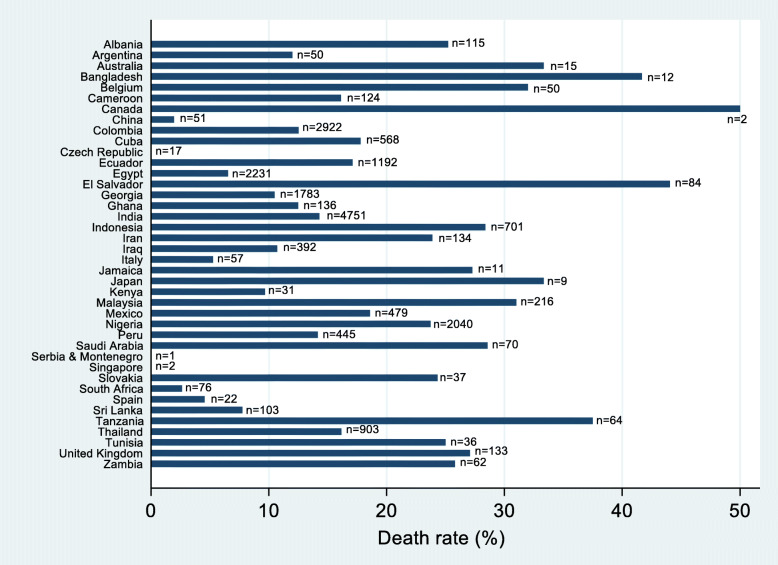
Percentage of patients in CRASH-2 countries that died within 4 weeks of randomisation; the number of patients randomised in each country is provided

Overall, 3076 patients died within 4 weeks, 1463 (14.5%) in the tranexamic acid group and 1613 (16.0%) in the control group. The crude death rate varied substantially across countries; for example, 23.7% (484/2040) patients died within 4 weeks in Nigerian trial centres, whilst centres contributing small number of patients, such as Serbia and Montenegro (*n* = 1), Singapore (*n* = 2) and Czech Republic (*n* = 17), had no deaths during the trial (Fig. 1).

### Random effects structure

Adjusting for baseline patient characteristics, there was significant variation in *underlying outcome* between countries and between centres within countries (Table 2). The ICC suggested that centres accounted for 16.4% of the total variation in death rates; 95% intervals in centre-specific odds ratio relative to the average in the same country ranged from 0.29 to 3.50, that is, the range is from approximately a third to three times the average odds of death in the same country. The corresponding ICC for countries, adjusted for patient characteristics and centre variance, suggested that country differences accounted for 6.0% of the variation in death rates; 95% intervals for the odds ratios ranged from 0.39 to 2.59 relative to the country average.

**Table 2:** Full results from final model

Covariate	Point estimate	Standard error	95% CI	*P* value
Treatment (active)	0.89	0.042	0.81 to 0.98	0.014
Time from injury (> 1–3 h)	0.86	0.054	0.76 to 0.97	0.015
Time from injury (> 3 h)	0.75	0.051	0.66 to 0.86	< 0.0001
Age (years)	0.99	0.007	0.98 to 1.01	0.332
Age^2^	1.00	0.00008	1.00 to 1.00	< 0.0001
SBP (mmHg)	0.93	0.004	0.93 to 0.94	< 0.0001
SBP^2^	1.00	0.00002	1.00 to 1.00	< 0.0001
GCS (moderate)	0.21	0.015	0.18 to 0.24	< 0.0001
GCS (mild)	0.07	0.004	0.06 to 0.08	< 0.0001
Injury type (penetrating)	0.81	0.054	0.71 to 0.93	0.002

Variance of country intercept: 0.236

Variance of centre intercept (country variance taken into account): 0.409

In contrast, there was no variation between centres or between countries in *treatment effect* estimates, with the variance between country random effects converging to zero.

**Fig. 2:**
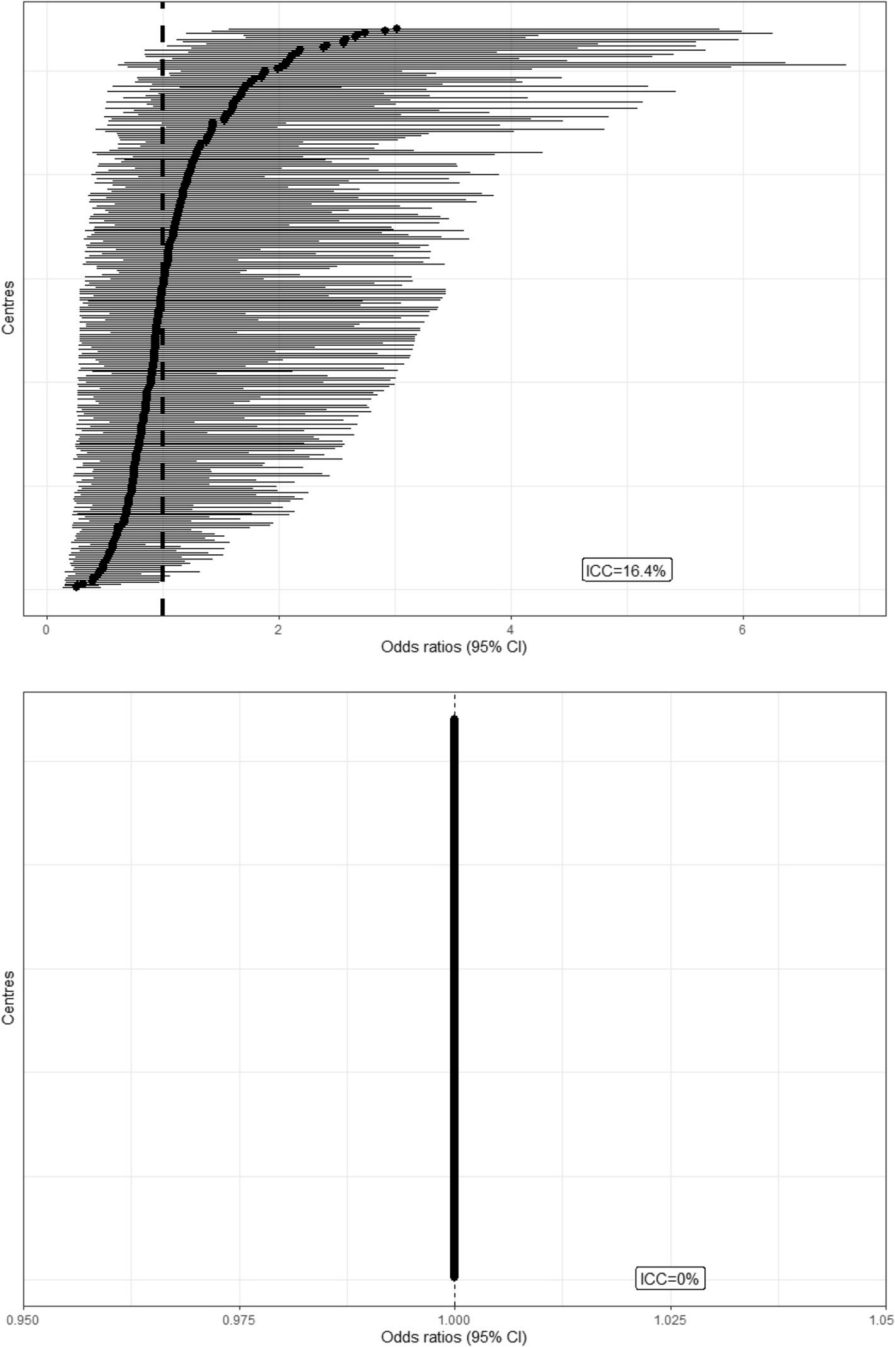
CRASH-2 centre-specific odds ratios (95% confidence intervals) for death in 4 weeks, relative to the average (top panel) and centre-specific treatment effects (95% confidence intervals), relative to the average (lower panel)

**Fig. 3:**
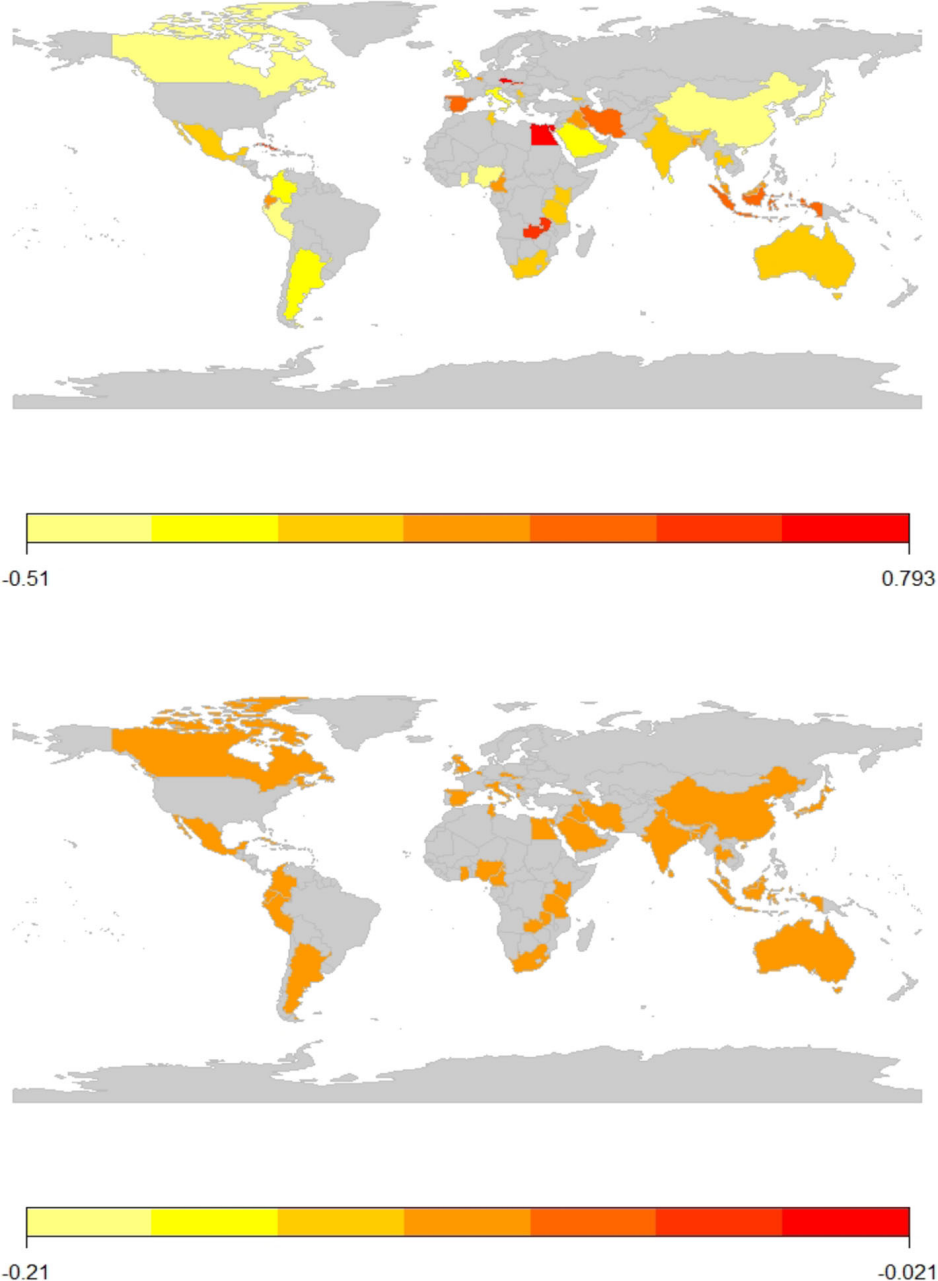
CRASH-2 country-specific odds ratios for death in 4 weeks, relative to the average (top panel) and country-specific treatment effects (95% confidence intervals), relative to the average (lower panel)

Figure 2 shows approximate odds ratios of death (relative to the average) for each centre in the top panel and the corresponding estimates for the odds ratios for treatment effect in the lower panel. In order to explore concerns about geographical variation in outcomes, in Fig. 3, participating countries are shaded according to their log (odds ratio) for death, relative to the average (top panel) and according to the log (odds ratio) for treatment effect (lower panel). This illustrates that countries with high and low odds of death appear to be distributed randomly across the world and that there is no variation in the effectiveness of tranexamic acid.

### Treatment and other fixed effects

The final model for all-cause mortality is given in the Additional file 1 and included SBP, SBP-squared, age, age-squared, GCS, time to treatment categories and injury type. Despite adjustment for these patient characteristics and for centre/country variation in outcomes, the point estimate of the treatment effect increases only slightly after adjustment for patient characteristics (Additional file 2: Table S1) and returns to the original estimate after further adjustment for between-country and centre variance. The statistical significance of the treatment effect decreases somewhat after adjusting for patient characteristics (*p* = 0.018), but increases after accounting for between centre and country variance.

### Explaining between-centre/country variance with centre/country level variables

One benefit of random effects models is the opportunity to assess centre/country-level variables associated with better and worse outcomes. In CRASH-2 mean age, proportion of cases with severe GCS, proportion with penetrating injuries and average time to treatment for each centre, as well as income level category for each country could be assessed (summary statistics in Table 3). The proportion of patients with severe GCS in a centre was associated with increased death rates. For every 10% increase in percentage of cases with severe GCS, the odds ratio was 11.5 (95% CI 6.37, 20.7) (Additional file 2: Table S2). Death rates were also higher in low- and middle-income countries relative to high-income countries (odds ratio 1.25 (1.13, 1.37)).

**Table 3:** Summary statistics for centre and country level characteristics in the CRASH-2 trial

Centre level characteristics	Mean (range)
Centre mean age (years)	36.3 (16.0–79.0)
GCS severe average centre proportion	0.23 (0–1)
GCS moderate average centre proportion	0.12 (0–1)
GCS mild average centre proportion	0.65 (0–1)
Time to treatment ≤ 1 h average centre proportion	0.06 (0–1)
Time to treatment 1–3 h average centre proportion	0.40 (0–1)
Time to treatment > 3 h average centre proportion	0.53 (0–1)
Penetrating injuries average centre proportion	0.25 (0–1)
Blunt* injuries average centre proportion	0.75 (0–1)
**Country level characteristics**	
Proportion in low/lower middle-income countries	0.51

Proportion in upper middle/high-income countries

0.49

### Investigating period effects

In order to assess whether patients recruited closer in time had more similar results, an exchangeable, four-level hierarchical model was fitted (patient within 6-month time period within centre within country). There was significant evidence that results varied by time period (*p* = 0.0002 for likelihood ratio test comparison with three-level model), although the variation in outcomes due to time period was only 2% of the total variation. Fitting time period as a fixed effect suggested that patients recruited later had lower odds of death. However, treatment effect was not related to period of recruitment in either model (See Additional file 2 for results).

## Discussion

### Summary of results

In our reanalysis of CRASH-2, accounting for the large between-centre and between-country variation did not change the estimated treatment effect for all-cause death within 4 weeks, although the statistical significance was slightly increased (*p* = 0.018 to *p* = 0.014). The reason for this is that the estimated variation in the effect of tranexamic acid between countries (Fig. 3), and between centres within countries (Fig. 2), is essentially zero. This is consistent with secondary analysis of the MRC CRASH trial [9], which investigated the effect of corticosteroids on death in 10,008 patients with head injuries. Lingsma et al. found that large between-centre differences in outcomes did not affect the estimate or statistical significance of the overall treatment effect in this trial [8].

Conversely, the forest plots (Fig. 2) and maps (Fig. 3) clearly illustrated variation in outcome without treatment by centre and by country respectively, after adjusting for patient baseline characteristics.

### Discussion and interpretation of our results

It is important to note that marginal models (generalised estimating equations) could have been used if the aim was only to adjust for clustering. However, random effects models were chosen as they allowed us to estimate contributions of variance from countries/centres and investigate influence of available covariates at centre and country level.

The reasons for variation in outcomes were not clear. At centre level, some variance in outcomes could be explained by the higher number of severe GCS cases in some centres, but the trial dataset did not include many centre-level covariates to allow detailed exploration. At the country level, outcomes were generally worse in low- and middle-income countries, but there was no clear geographical pattern, with lower than average odds of death evident in countries as diverse as China, Kenya and Canada. Moreover, CRASH-2 did not contain detailed information on country-specific health service provision and infrastructure. We note that countries (or centres) with a small number of patients in the trial tended to have extreme results (very high or low death rates). However, small centres may suffer from small sample bias (tendency to have extreme outcomes by chance), so that care has to be taken when interpreting these figures.

In CRASH-2, the effect of tranexamic acid did not vary across centres. Gruen et al. raised concerns about generalisability of CRASH-2 trial results applied to high-income settings, since most trial participants were treated in low- and middle-income countries [6]. This seems to conflate variation in outcomes (independent of treatment) and variation in treatment effects. In a response, Roberts and Prieto-Marino illustrated how two centres could have very different risks of death in the control group, but the relative risks comparing the treatment group and control group within the centres can still be the same [10]. The biological mechanism by which tranexamic acid works (stopping clot breakdown and reducing heavy bleeding) is not expected to vary between patients (and therefore centres), so that results due to bleeding-related deaths are generalizable, although deaths from other causes may differ. The trial treatment was a single dose of tranexamic acid or matched placebo, so that there was little variation in delivery of treatment and selection bias was avoided. Our reanalysis of CRASH-2 supported the homogeneity of treatment effect on death at 4 weeks across countries and centres, including high-income countries.

Variation in the outcome or treatment effect can depend on the choice of statistical model [11]. However, our results were almost identical whether our analysis used odds ratios (from logistic regression) or risk ratios (from Poison regression) to describe treatment effects. We note that this result may not generalisable to (open label) trials of more complex interventions, such as surgery or mental health interventions, where the treatment delivery may depend on the health provider [12].

Studies of complex surgical interventions have reported changes in outcomes as a trial progresses [13]. Hence, studies may warrant investigation of a ‘period effect’, where patients recruited closer in time have more similar results due to protocol changes, learning effects or trial experience. In the CRASH-2 trial, time period explained 2% of the total variation in outcome, but it was statistically significant at traditional levels, with later patients having lower odds of death. This was unexpected in this double-blind trial of a single episode treatment and may result from a general shift towards recruiting slightly lower-risk patients over time, as experience in the trial increases. The treatment effect was not related to period of recruitment.

### How it fits with literature/guidelines

Currently, there is no consensus on when and how centre variance should be adjusted for in multicentre trials. The ICH-E9 guideline on multicentre trials focuses on possible heterogeneity of treatment effect and on avoiding having few subjects in some centres [14]. For trials with positive treatment effects and appreciable numbers of patients per centre, it recommends an exploratory analysis to identify any heterogeneity of treatment effects across centres and to assess its impact on generalisability of results. It does not strongly suggest that centre differences in outcomes must be accounted for in primary trial analysis. ICH-E9 guidelines [14] also discuss the use of random effects models for exploring heterogeneity of the treatment effects, suggesting that centre effects on outcome and treatment effects are especially relevant when the number of sites is large. Including centres as fixed effects is appropriate if the number of centres in the trial is small or if interest surrounds estimation of centre effects themselves (see for example, Rabe-Hesketh and Skrondal [15]). However, if the number of centres is large, with some recruiting only a few patients, or if centres are viewed as a sample of the wider population of centres that might benefit from treatment, but are not of interest themselves, a random effects model is more appropriate.

Simulation studies have been carried out to look at any change in power when accounting for between-centre variation. Kahan and Morris found that type 1 error rates became large when between centre variation or clustering was ignored for both continuous and binary outcomes [4, 16]. Our own simulation studies based on data with the same structure as CRASH-2 also found that, for binary outcomes, type 1 error rates were substantially inflated when between-centre variance was ignored in the analysis, particularly if centres had unequally sized treatment allocation.

### Strengths and weaknesses

The large sample size in the CRASH-2 trial and the small amount of missing data meant that there was high power and small chance of bias in estimation of the overall treatment effect. The large number of centres and countries also allows reliable estimation of the distribution of outcomes and treatment effects. Thus, we can be confident in conclusions around centre variation and (lack of) treatment effect heterogeneity by country. A limitation was the lack of centre and country level variables in the CRASH-2 dataset, which could have helped explain geographical and health service factors that contribute to different outcomes.

## Conclusions

In conclusion, in multicentre studies with binary outcome, clustering should be accounted for to maximise power and reduce bias in treatment effect estimates and standard errors. Randomisation should be stratified by centre where possible; if not possible, analysis should adjust for clustering by centre. Random effects models are an efficient way to do this when the number of centres is large (≥ 20), treatment assignments are unequal within centres and where there are few patients per centre. We recommend that care should be taken when interpreting between-centre variation, in particular recognising that the treatment effect is not likely to vary by country or income setting if there is an established biological mechanism. However, the underlying risk of death is likely to vary across different settings.

## Supplementary Information

**Additional file 1.** Details of statistical models.

**Additional file 2: Table S1.** Average treatment effect for all-cause deaths at 4 weeks in the CRASH-2 trial. **Table S2.** Effect of within and between centre-level covariates in the CRASH-2 trial. **Table S3.** Results of the 4-level random effect model investigating period effect with added random intercept for 6 month block within site. **Table S4.** Results of the 3-level model with fixed effect for 6 month block.

## Data Availability

The dataset from the CRASH-2 (2010) trial can be found at https://freebird.lshtm.ac.uk.
