# Supplementary material for: Including random centre effects in design, analysis and presentation of multi-centre trials
Source: Trials. 2021 May 22;22:357. doi: 10.1186/s13063-021-05266-w (PMC8140487; doi:10.1186/s13063-021-05266-w)
Supplement: Supplementary file 1 — Additional file 1. Details of statistical models. [file 13063_2021_5266_MOESM1_ESM.docx]

**Additional file 1 Details of statistical models**

***Derivation of final mixed effects model***

Below are the assumptions and formulation for the saturated model considered initially.

$Y_{ijk}$ is the outcome for patient $i$ in centre $j$ in country $k$, with $Y_{ijk}=1$ if the patient dies within 28 days of injury and 0 otherwise. $Y_{ijk}$ has distribution:

$$Y_{ijk} \sim Bernoulli(\lambda_{ijk})$$

with $\lambda$ the mean number of events. The formulation for the logistic mixed model is:

$$logit \left( \lambda_{ijk} \right|\beta_{0}, \beta_{1}, \boldsymbol{\beta}_{\boldsymbol{2}}, u_{0jk} , v_{0k}, u_{1jk} , v_{1k}) = \beta_{0} + u_{0jk}+ v_{0k}+\left( \beta_{1}+ u_{1jk}+ v_{1k} \right) T_{ijk}+ \boldsymbol{\beta}_{\boldsymbol{2}}^{T}\boldsymbol{X}_{ijk}$$

where:

- $T_{ijk}=1$ if the patient is assigned to the active treatment and 0 otherwise.
- $\boldsymbol{X}_{ijk}$is vector of baseline patient characteristics.
- $\beta_{0}$ is the fixed intercept, $\beta_{1}$ is the fixed treatment effect, and $\boldsymbol{\beta}_{2}$ is a vector of coefficients for baseline patient characteristics.
- $u_{0jk}$ is the random intercept which varies by centre within country, $u_{1jk}$ is the random coefficient for treatment which varies by centre within country,
- $v_{0k}$ is the random intercept which varies by country, and $v_{1k}$ is the random coefficient for treatment which varies by country.

These random effects have distributions:

$$\left[ \begin{matrix} u_{0jk} \\ u_{1jk} \end{matrix} \right] | v_{0k} , v_{1k} , T_{ijk}, X_{ijk} \sim N\left[ \left( \begin{matrix} 0 \\ 0 \end{matrix} \right), \left( \begin{matrix} \sigma_{u0}^{2} & \sigma_{u01} \\ \sigma_{u10} & \sigma_{u1}^{2} \end{matrix} \right) \right]$$

$$\left[ \begin{matrix} v_{0k} \\ v_{1k} \end{matrix} \right] | T_{ijk}, X_{ijk} \sim N\left[ \left( \begin{matrix} 0 \\ 0 \end{matrix} \right), \left( \begin{matrix} \sigma_{v0}^{2} & \sigma_{v10} \\ \sigma_{v10} & \sigma_{v1}^{2} \end{matrix} \right) \right]$$

An exchangeable covariance structure for the random effects was assumed with independence between intercepts and coefficients ($\sigma_{v10}=\sigma_{v10}=\sigma_{u01}=\sigma_{u10}=$0). This means the random intercept was assumed to be uncorrelated with the random treatment coefficient, for centres and countries. This was assumed because, in the example of between-country variation, it is unlikely that (adjusting for patient characteristics) the countries will vary in outcome in a related way to the way treatment effect varies by country. Delivery and biological action of a drug like tranexamic acid does not vary appreciably, and countries have a baseline event rate which is separate from the treatment effect.

For the level 1 errors $\epsilon_{ijk}$the variance is assumed to be $\sigma_{e}^{2}=$ $\frac{\pi^{2}}{3}$, reflecting the assumption of a latent (unobserved) continuous variable, with a positive response (event) observed only when this variable exceeds some threshold. The observed binary response can be thought of as a categorization of the unobservable latent variable, above and below the threshold. If the latent variable has a standard logistic distribution with mean zero and variance $\frac{\pi^{2}}{3}$ , it can be shown analytically that the relationship between the outcome and the latent variable results in a logistic regression model for the probability of a positive response^15^.

Initially, to investigate the random effects structure, this saturated model included all available baseline variables, along with two-way interactions with treatment and squared terms for continuous variables. Random effects were assessed for removal from the model in the following order:

1. Country treatment coefficient $v_{1k}$,
2. Country intercept $v_{0k}$,
3. Centre treatment coefficient $u_{1jk}$,
4. Centre intercept $u_{0jk}$.

Centres with and without each random effect term were compared using likelihood ratio tests holding the fixed effects structure constant. Once the random effects structure was determined, the fixed effects structure was simplified using stepwise removal of non-significant variables based on Wald statistics.

Empirical Bayes estimates of random effects were retrieved and approximate 95% centre/country ranges of odds ratios for death in each country, or for each centre within country compared to the average were calculated as $[exp(-1.96*\sigma_{v0}$) , $exp(1.96* \sigma_{v0}$)] and $[exp(-1.96* \sigma_{u0}$) , $exp(1.96* \sigma_{u0}$)] respectively.

***Intra-class correlation coefficients***

The intra-class correlation coefficient for patients who share a country but not centre (after adjusting for covariates) is estimated as:

ICC = $\frac{\sigma_{v0}^{2}}{\sigma_{v0}^{2}+ \sigma_{u0}^{2}+ \sigma_{e}^{2}}$

and patients from the same centre (and therefore same country):

ICC = $\frac{\sigma_{v0}^{2}+ \sigma_{u0}^{2}}{\sigma_{v0}^{2}+ \sigma_{u0}^{2}+ \sigma_{e}^{2}}$

***Structure of the final model***

The final model had the form:

$$logit \left( \lambda_{ijk} \right|u_{0jk} , v_{0k})= \beta_{0}+u_{0jk}+ v_{0k}+ \beta_{1} . T_{ijk}+ \beta_{2}. {SBP}_{ijk}+ \beta_{3}. {age}_{ijk}+ \beta_{4}. {age}_{ijk}^{2} + \beta_{5}. {SBP}_{ijk}^{2}+ \beta_{6}. {Moderate GCS}_{ijk}+ \beta_{7}. {Mild GCS}_{ijk}+ \beta_{8}. \left[ \leq1 hour from injury \right]_{ijk} + \beta_{9}. [{>1-3 hours from injury]}_{ijk}+ \beta_{10}.{Penetrating injury}_{ijk}$$

***Centre-level covariates***

Centre-level covariates were added to the random effects model individually to see whether they accounted for any of the between-centre variance. They were added in the following way to distinguish the within centre ($\beta_{w})$and between centre effects ($\beta_{b})$ of these variables.

$$logit \left( \lambda_{ijk} \right|u_{0jk} , v_{0k})= \beta_{0}+u_{0jk}+ v_{0k}+\ldots+{\beta_{b}(Mean centre age}_{jk})$$

$$+ \beta_{w}({Mean centre age}_{jk}- {Patient age}_{ijk})$$
