# Supplementary material for: Including random centre effects in design, analysis and presentation of multi-centre trials
Source: Trials. 2021 May 22;22:357. doi: 10.1186/s13063-021-05266-w (PMC8140487; doi:10.1186/s13063-021-05266-w)
Supplement: Supplementary file 2 — Additional file 2: Table S1. Average treatment effect for all-cause deaths at 4 weeks in the CRASH-2 trial. Table S2. Effect of within and between centre-level covariates in the CRASH-2 trial. Table S3. Results of the 4-level random effect model investigating period effect with added random intercept for 6 month block within site. Table S4. Results of the 3-level model with fixed effect for 6 month block. [file 13063_2021_5266_MOESM2_ESM.docx]

**Additional file 2 Additional tables and Figures**

**Table S1.** Average treatment effect for all-cause deaths at 4 weeks in the CRASH-2 trial

| Analysis | Model | Odds ratio | Standard error | 95% Confidence interval | P-value |
| --- | --- | --- | --- | --- | --- |
| Unadjusted | Fixed effect logistic regression | 0.89 | 0.035 | 0.83 to 0.96 | 0.004 |
| Adjusted for patient characteristics* | Fixed effect logistic regression | 0.90 | 0.041 | 0.82 to 0.98 | 0.018 |
| Adjusted for patient characteristics, between country differences, and between centre- within-country differences | Mixed effect logistic regression model with random intercepts for country and centre | 0.89 | 0.042 | 0.81 to 0.98 | 0.014 |

*SBP, SBP^2^, GCS, time from injury to treatment, type of injury, age, age^2^

**Table S2**: Effect of within and between centre-level covariates in the CRASH-2 trial

|  | Odds ratio* | 95% Confidence interval | P-value |
| --- | --- | --- | --- |
| Mean centre age  **(between centre effect)** | 1.01 | 0.98 to 1.03 | 0.641 |
| Age – mean centre age  **(within centre effect)** | 1.02 | 1.02 to 1.03 | <0.0001 |
| Test equality of these coefficients | p=0.1023 | | |
|  | | | |
| GCS Severe centre proportion | 11.5 | 6.37 to 20.7 | <0.0001 |
| GCS Severe within centre effect | 10.6 | 9.46 to 11.9 | <0.0001 |
| Test equality of these coefficients | p=0.7921 | | |
|  | | | |
| GCS Moderate centre proportion | 0.32 | 0.11 to 0.93 | 0.036 |
| GCS Moderate within centre effect | 1.01 | 0.89 to 1.14 | 0.870 |
| Test equality of these coefficients | p=0.0359 | | |
|  | | | |
| Penetrating injury centre proportion | 1.00 | 0.54 to 1.85 | 0.993 |
| Penetrating injury within centre effect | 0.81 | 0.71 to 0.92 | 0.001 |
| Test equality of these coefficients | p=0.4924 | | |
|  | | | |
| Between 1-3 hours since injury centre proportion | 0.91 | 0.54 to 1.51 | 0.709 |
| Between 1-3 hours since injury within centre effect | 1.00 | 0.90 to 1.11 | 0.996 |
| Test equality of these coefficients | p=0.7072 | | |
|  | | | |
| Over 3 hours since injury | 1.16 | 0.73 to 1.82 | 0.533 |
| Over 3 hours since injury within centre effect | 0.81 | 0.72 to 0.90 | <0.0001 |
| Test equality of these coefficients | p=0.1330 | | |
|  | | | |
| Low to lower middle income country | 1.25 | 1.13-1.37 | <0.0001 |

*each analysis of centre variable was done separately and adjusted for the remaining patient level characteristics

**Table S3: Results of the 4-level random effect model investigating period effect with added random intercept for 6 month block within site**

| Covariate | Odds ratio | Standard error | 95% CI | P-value |
| --- | --- | --- | --- | --- |
| Treatment (active) | 0.89 | 0.041 | 0.81 to 0.97 | 0.009 |
| Time from injury (>1-3 hours) | 0.86 | 0.054 | 0.76 to 0.97 | 0.014 |
| Time from injury (>3 hours) | 0.74 | 0.050 | 0.65 to 0.85 | <0.0001 |
| Age (years) | 1.00 | 0.007 | 0.98 to 1.02 | 0.614 |
| Age^2^ | 1.00 | 0.00008 | 1.00 to 1.00 | <0.0001 |
| SBP (mmHg) | 0.97 | 0.002 | 0.97 to 0.98 | <0.0001 |
| SBP^2^ | 1.00 | 0.00001 | 1.00 to 1.00 | <0.0001 |
| GCS (Moderate) | 0.21 | 0.015 | 0.18 to 0.24 | <0.0001 |
| GCS (Mild) | 0.06 | 0.004 | 0.06 to 0.07 | <0.0001 |
| Injury type (penetrating) | 0.82 | 0.053 | 0.72 to 0.93 | 0.002 |

Variance of country intercept: 0.217

Variance of centre intercept (country variance taken into account): 0.393

Variance of 6 month block intercept (country and centre variance taken into account): 0.079

ICC for block: 0.02 (2%)

**Table S4: Results of the 3-level model with fixed effect for 6 month block**

| Covariate | Odds ratio | Standard error | 95% CI | P-value |
| --- | --- | --- | --- | --- |
| Treatment (active) | 0.88 | 0.041 | 0.81 to 0.97 | 0.007 |
| Time from injury (>1-3 hours) | 0.85 | 0.053 | 0.76 to 0.97 | 0.011 |
| Time from injury (>3 hours) | 0.74 | 0.049 | 0.65 to 0.84 | <0.0001 |
| Age (years) | 1.00 | 0.007 | 0.98 to 1.01 | 0.634 |
| Age^2^ | 1.00 | 0.00008 | 1.00 to 1.00 | <0.0001 |
| SBP (mmHg) | 0.97 | 0.002 | 0.97 to 0.98 | <0.0001 |
| SBP^2^ | 1.00 | 0.00001 | 1.00 to 1.00 | <0.0001 |
| GCS (Moderate) | 0.21 | <0.0001 | 0.19 to 0.25 | <0.0001 |
| GCS (Mild) | 0.07 | 0.004 | 0.06 to 0.08 | <0.0001 |
| Injury type (penetrating) | 0.82 | 0.053 | 0.72 to 0.93 | 0.003 |
| 6-month block |  | | | |
| 1 | - | - | - | - |
| 2 | 0.82 | 0.128 | 0.60 to 1.11 | 0.204 |
| 3 | 0.70 | 0.105 | 0.52 to 0.94 | 0.018 |
| 4 | 0.77 | 0.111 | 0.58 to 1.02 | 0.068 |
| 5 | 0.82 | 0.117 | 0.62 to 1.08 | 0.158 |
| 6 | 0.61 | 0.086 | 0.46 to 0.80 | <0.0001 |
| 7 | 0.62 | 0.087 | 0.47 to 0.81 | 0.001 |
| 8 | 0.63 | 0.090 | 0.48 to 0.83 | 0.001 |
| 9 | 0.62 | 0.089 | 0.47 to 0.82 | 0.001 |

Variance of country intercept: 0.215

Variance of centre intercept (country variance taken into account): 0.428
